# A rare coding mutation in the MAST2 gene causes venous thrombosis in a French family with unexplained thrombophilia: The Breizh MAST2 Arg89Gln variant

**DOI:** 10.1371/journal.pgen.1009284

**Published:** 2021-01-19

**Authors:** Pierre-Emmanuel Morange, Franck Peiretti, Lenaick Gourhant, Carole Proust, Omar Soukarieh, Anne-Sophie Pulcrano-Nicolas, Ganapathi-Varma Saripella, Luca Stefanucci, Romaric Lacroix, Manal Ibrahim-Kosta, Catherine A. Lemarié, Mattia Frontini, Marie-Christine Alessi, David-Alexandre Trégouët, Francis Couturaud

**Affiliations:** 1 Aix Marseille Univ, INSERM, INRAE, C2VN, Marseille, France; 2 Hematology laboratory, CHU Timone, Marseille, France; 3 EA3878-GETBO, Univ Brest, Department of internal medicine and chest diseases, FCRIN_INNOVTE, CHU Brest, Brest, France; 4 INSERM U1078, Brest, France; 5 Sorbonne Université, UPMC, INSERM UMR_S 1166, Paris, France; 6 INSERM UMR 1219, Bordeaux Population Health Research Center, University of Bordeaux, France; 7 National Institute for Health Research BioResource, Cambridge University Hospitals, Cambridge Biomedical Campus, Cambridge, United Kingdom; 8 NHS Blood and Transplant, Cambridge Biomedical Campus, Cambridge, United Kingdom; 9 British Heart Foundation Centre of Excellence, Cambridge Biomedical Campus, United Kingdom; Universitatsklinikum Hamburg-Eppendorf, GERMANY

## Abstract

Rare variants outside the classical coagulation cascade might cause inherited thrombosis. We aimed to identify the variant(s) causing venous thromboembolism (VTE) in a family with multiple relatives affected with unprovoked VTE and no thrombophilia defects. We identified by whole exome sequencing an extremely rare Arg to Gln variant (Arg89Gln) in the Microtubule Associated Serine/Threonine Kinase 2 (*MAST2*) gene that segregates with VTE in the family. Free-tissue factor pathway inhibitor (f-TFPI) plasma levels were significantly decreased in affected family members compared to healthy relatives. Conversely, plasminogen activator inhibitor-1 (PAI-1) levels were significantly higher in affected members than in healthy relatives. RNA sequencing analysis of RNA interference experimental data conducted in endothelial cells revealed that, of the 13,387 detected expressed genes, 2,354 have their level of expression modified by MAST2 knockdown, including *SERPINE1* coding for PAI-1 and *TFPI*. In HEK293 cells overexpressing the MAST2 Gln89 variant, *TFPI* and *SERPINE1* promoter activities were respectively lower and higher than in cells overexpressing the MAST2 wild type. This study identifies a novel thrombophilia-causing Arg89Gln variant in the *MAST2* gene that is here proposed as a new molecular player in the etiology of VTE by interfering with hemostatic balance of endothelial cells.

## Author summary

Venous thromboembolism (VTE) is a multifactorial disease in which the genetic burden is high. We here present the case of a French family with multiple relatives affected with unprovoked VTE (i.e. that occurred in the absence of clinical risk factors) in which no thrombophilia defects had been identified. Adopting a whole exome sequencing approach, we identified an extremely rare variant located in the Microtubule-associated serine/threonine-protein kinase-2 (*MAST2*) gene that perfectly segregates with the VTE phenotype and that interferes with hemostatic balance of endothelial cells. Our results pave the way for adding *MAST2* to the list of genes to be sequenced and looked for in thrombophilia families with unprovoked VTE.

## Introduction

Venous thromboembolism (VTE) is a multifactorial disease in which the genetic burden can be characterized by a sibling relative risk of ~2.5 [1] and an estimated heritability between 35%-60% [2]. As for many multifactorial diseases, the spectrum of genetic factors contributing to VTE susceptibility ranges from common single nucleotide polymorphisms (SNPs) associated with low-to- moderate genetic effects to private variants segregating within families and associated with very high relative risk of disease. About thirty common SNPs with minor allele frequency (MAF) greater than ~1% have so far been reported to associate with the risk of VTE in the general population, each of them being characterized by an Odds Ratio for disease ranging between 1.06 and 3.0 [3]. Uncommon genetic variants with MAF between 0.1% and 1% have also been reported such as the *THBD* c.-151G>T variant [4] or protein S Heerlen [5]. At the extreme low frequency side of the genetic spectrum reside private variants (frequencies < 1‰) that are generally 'loss of function' variants associated with at least a 10-fold increased risk in heterozygote individuals. These variants mainly affect the coagulation cascade through inherited deficiencies of the three main natural anticoagulants, antithrombin, protein C and protein S. However, rare variants outside the classical coagulation cascade have also been proposed to cause severe rare inherited thrombosis generally referred to as inherited thrombophilia [6]. Their identification has been facilitated by the development of whole exome/genome sequencing technologies.

Here we present the case of a French family with multiple relatives affected with unprovoked VTE (i.e. VTE that occurred in the absence of clinical risk factors) in which no thrombophilia defects had been identified. Adopting a whole exome sequencing (WES) approach, we identified an extremely rare variant located in the Microtubule-associated serine/threonine-protein kinase-2 (MAST2) gene that perfectly segregates with the VTE phenotype. Complementary RNA sequencing (RNA-seq) experiments performed in endothelial cells treated with siRNAs targeting MAST2 and gene reporter experiments demonstrate, for the first time, a role of MAST2 in thrombotic processes in particular through its involvement in the regulation of *TFPI* and *SERPINE1* genes.

## Results

### Pattern of coagulation parameters in the studied family

**Fig 1:**
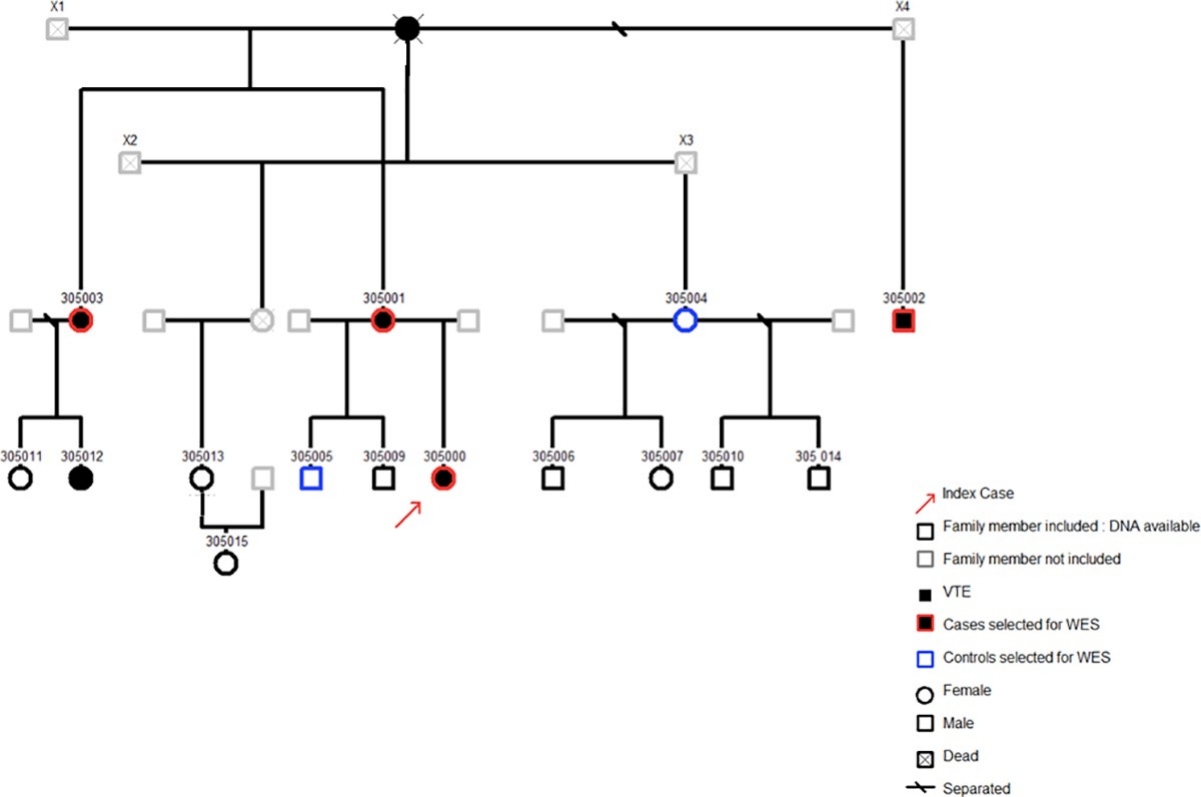
Genealogical tree of the family.

**Fig 2:**
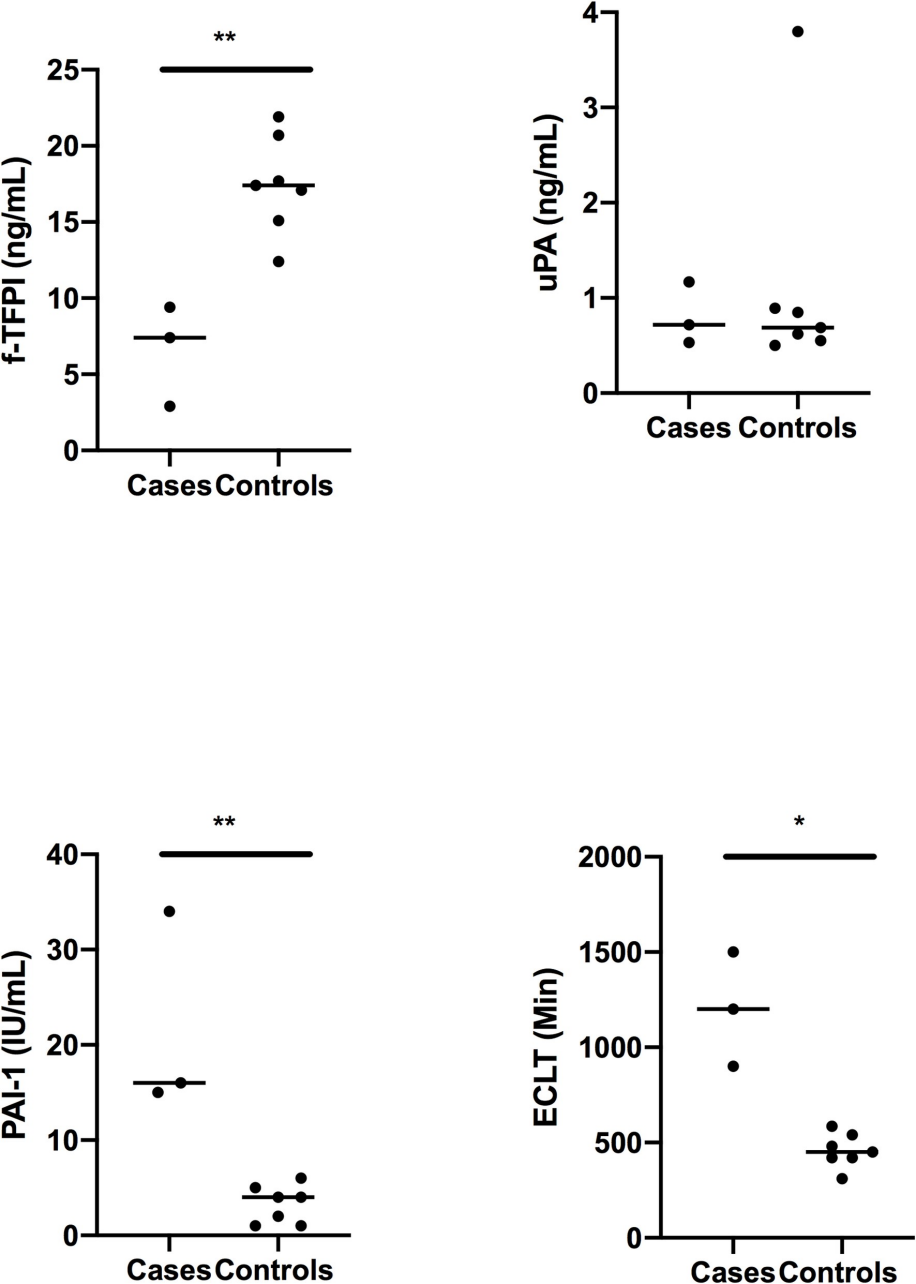
Free-TFPI (f-TFPI), euglobulin clot lysis time (ECLT), Plasminogen Activator Inhibitor-1 (PAI-1) and Urokinase (UPA) plasma levels in VTE cases and controls from the family. Statistical analyses were made using Mann-Whitney. * p<0.05 and **p<0.01: significant vs controls.

The genealogical tree of the family with unknown thrombophilia is reported in Fig 1. Measurement of coagulation parameters in family members are reported in Table 1. While differences in prothrombin time, International Normalized Ratio (INR), activated partial thromboplastin time, Factor II (FII), and Factor X (FX) were due to the use of antivitamin K in cases, unexpected nominal statistical differences (p < 0.05) were observed for plasma levels of the anticoagulant protein free-Tissue Factor Pathway Inhibitor (f-TFPI) and the antifibrinolytic protein Plasminogen Activator Inhibitor-1 (PAI-1). Plasma levels of f-TFPI were significantly (p = 0.01) lower in the affected family members compared to healthy relatives (6.6 +/- 1.9 ng/mL vs 17.4 +/- 1.2 ng/mL) (Fig 2) while the opposite pattern was observed for PAI-1 plasma levels (21.7 +/- 6.1 IU/mL vs 3.3 +/-0.7 IU/mL, p = 0.01) (Fig 2).

**Table 1:** Measurement of coagulation parameters in the 10 subjects with citrated plasma available.

Subjects	Status	PT (%)	INR (ratio)	aPTT (sec)	Fibrinogen (g/L)	FII (%)	FV (%)	FX (%)	f-TFPI (ng/mL)	PAI-1 (UI/mL)
305000	case	30	2.55	49.5	4.16	27	64	13	7.4	15
305001	case	26	2.88	46.7	4.3	18	76	9	2.9	16
305002	case	33	2.29	40.1	3.87	29	96	14	9.5	34
305004	control	98	1.01	32.4	4.93	99	86	105	17.2	1
305006	control	93	1.04	39	1.97	82	91	96	17.7	4
350007	control	100	1	35.4	2.62	107	75	122	15.1	1
305010	control	114	0.93	34.3	3.17	103	117	102	17.1	2
305013	control	104	0.98	35.1	3.13	97	95	90	21.9	4
305014	control	98	1.01	33.9	2.66	102	102	98	20.7	5
305015	control	106	0.97	37.8	3.26	95	91	95	12.3	6

PT: prothrombin Time; INR: International Normalized Ratio; aPTT: activated partial thromboplastin time; FII: Factor II; FV: Factor V; FX: Factor X; f-TFPI: free-Tissue Factor Pathway Inhibitor; PAI-1: Plasminogen activator inhibitor-1

### Identification of a MAST2 variant following the WES strategy

As the familial transmission of the disease was compatible with an autosomal dominant inheritance, we first selected variants (n = 14,406) shared by all cases and then excluded those that were carried by controls, leaving 1,153 variants as potential candidates. Among those, 108 were likely functional variants (stop loss/stop gain, frameshift insertion/deletion, non-synonymous and splicing) that we selected for further investigations. Then bioinformatics search in public genomic data repositories identified 2 as very rare candidates. One, rs151257275, was a nonsynonymous Arg to Gln variant in the *ADAMTS10* gene, with allele frequency ~0.005 and predicted to have no deleterious effect on the protein function. The second, rs1387081220 is a nonsynonymous Arg to Gln mutation (Arg89Gln) in exon 2 of the *MAST2* gene and it has 2 independent heterozygous cases reported in gnomAD[7].

These two candidate variants were then genotyped in all individuals, including those used for WES and their relatives with available DNA that were not part of the WES analysis (1 case and 8 controls). Only the *MAST2* variant perfectly co-segregated with the VTE phenotype whereas one case (305012) did not carry the *ADAMTS10* variant indicating that this variant should no longer be a candidate (S1 Table).

We genotyped the uncharacterized *MAST2* variant in 6,790 VTE cases and 5,970 healthy individuals and did not detect additional carriers providing strong argument that this variant was extremely rare. The look-up performed in other large cohort studies (i.e. 100,000 Genomes Project, gnomAD, NIHR BioResource-Rare Diseases, H3-Africa and GenomeAsia 100K; S2 Table, which includes relevant references) showed that the MAST2 Arg89Gln variant was only present in 2 alleles out of the ~ 345,000 total sequences in these projects (S2 Table).

*In silico* prediction tools considered this variation to be of strong deleteriousness (SIFT: deleterious, Polyphen: probably damaging, CADD score ranging from 27 to 34 according to reference database/versions). However, the predicted functional impact of the variant is rather unknown. It is not estimated to affect splicing mechanisms as assessed by QUEPASA, HEXplorer and HAL softwares [8]. The variant is located in a very conserved region and affects amino acid (AA) 89 in the exon 2 of the gene, outside the two known protein domains of the molecule, a Protein Kinase domain ranging from 512AA to 854AA and a PDZ domain from 1104AA to 1192AA.

### Functional characterization of the mutation and its structural gene

**Fig 3:**
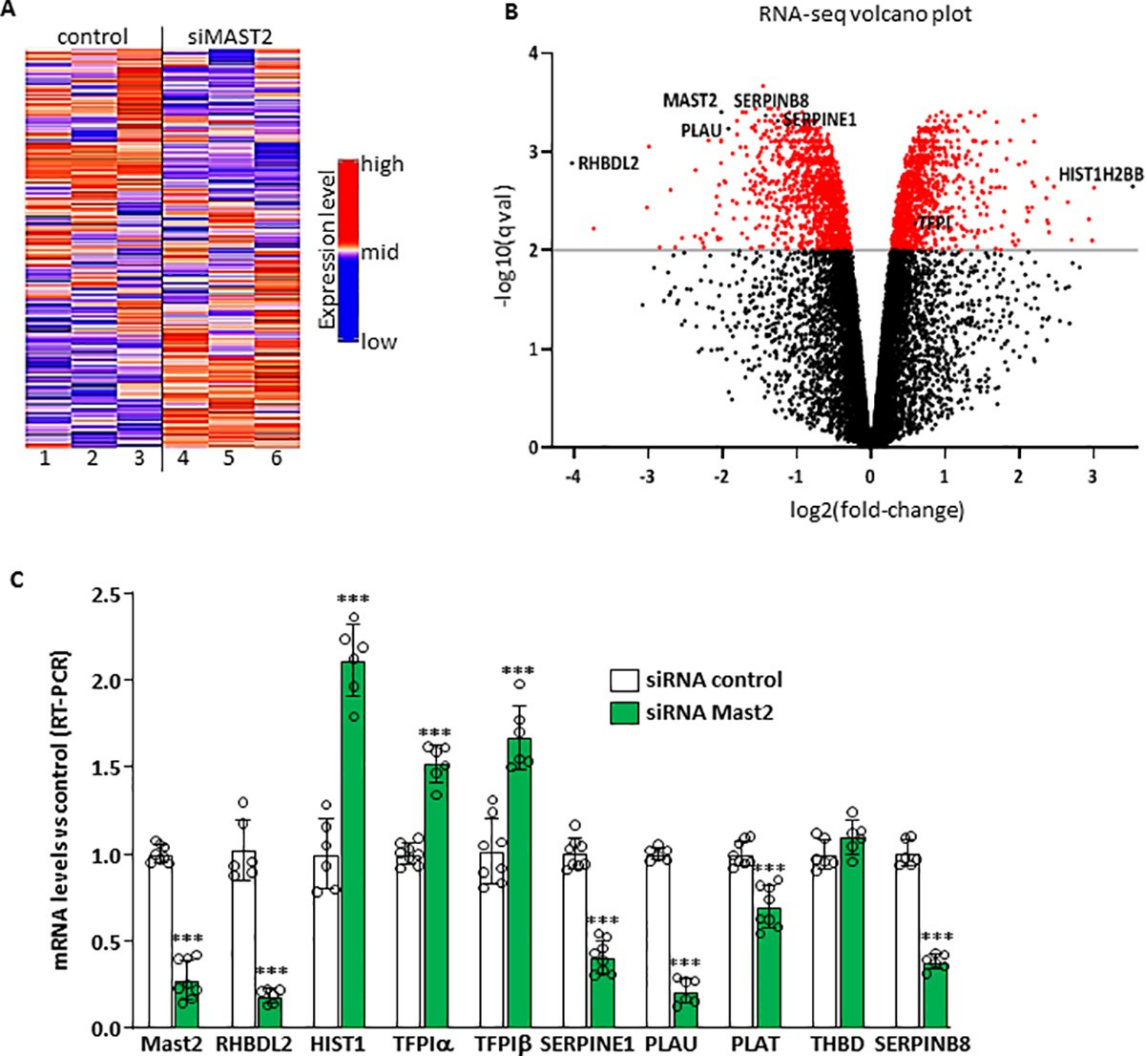
(A) Heatmap of the 5000 genes most differently expressed between cells treated with control siRNA (lanes 1–3) and with MAST2 specific siRNA (lanes 4–6). (B) volcano plot. (C) Validation of the RNA-seq. ECV304 cells were transfected with control siRNA or with two MAST2 specific siRNA. 48 hours post transfection, mRNA levels of MAST2, RHBDL2, HIST1H2BB (HIST1), TFPIα and β, SERPINE1, PLAU, PLAT, THBD and SERPINB8 were analyzed by RT-PCR. Data are means ± Standard Deviation. Statistical analyses were made using unpaired t-test. ***p<0.001: significant vs control.

The observation that plasma concentrations of f-TFPI and PAI-1 (two proteins produced by the endothelium) were significantly associated with the presence of the MAST2 Arg89Gln variant, led us to propose the involvement of MAST2 in regulating some endothelial properties. To gain a deeper understanding of the impact of this critical, yet relatively unknown contributor of endothelial homeostasis, an RNA-Seq based analysis was performed to compare the mRNA expression profiles of ECV 304 endothelial cells, with those of *MAST2* knockdown cells. The heatmap (Fig 3A) and volcano plot (Fig 3B) showed global expression changes in MAST2 knockdown cells. The full list of differential association p-value is given in S3 Table. Of the 13,387 expressed genes that have been detected, 2,354 have their expression modified (FDR < 1%) by *MAST2* knockdown. As expected, *MAST2* was one of the genes which expression was the most significantly reduced (ranked 8 in terms of differential association p-value; log2 fold change (log2FC) ~ -2). The expression of *RHBLD2* was the most reduced (ranked 689; log2FC– 4.3) and that of *HIST1H2BB* the most increased (ranked 897; log2FC 3.53) by *MAST2* knockdown. The expression of some genes known to regulate the hemostatic properties of endothelial cells was altered by *MAST2* knockdown. This was the case for *SERPINE1* (coding for PAI-1) (ranked 42; log2FC ~ -1.26), *PLAU* (coding for urokinase) (ranked 85; log2FC -2), *SERPIN B8* (ranked 22; log2FC 1.42) (a serine protease inhibitor hypothesized to co-express with PLAU as evidenced by text mining search tools, see Materials and Methods), and *TFPI* (ranked 1697; log2FC ~0.60). The expression of *PLAT* (coding for tissue plasminogen activator, t-PA) and *THBD* (coding for thrombomodulin), two genes also involved in the regulation of endothelial function, was not significantly altered.

Real-time PCR analyses (Fig 3C) confirmed most of the RNA-Seq findings observed for aforementioned genes. Treatment of endothelial cells with siRNA specific for *MAST2* decreased the expression of *MAST2*, *RHBDL2*, *SERPINE1*, *PLAU* and increased the expression of *HIST1H2BB*, *TFPIα* and *TFPIβ*. *PLAT* expression was also observed to be significantly decreased, but to a smaller extent, and *THBD* expression was not significantly modified (Fig 3C). These results underline the ability of *MAST2* to regulate mRNA levels of genes encoding proteins involved in coagulation/fibrinolysis cascades.

**Fig 4:**
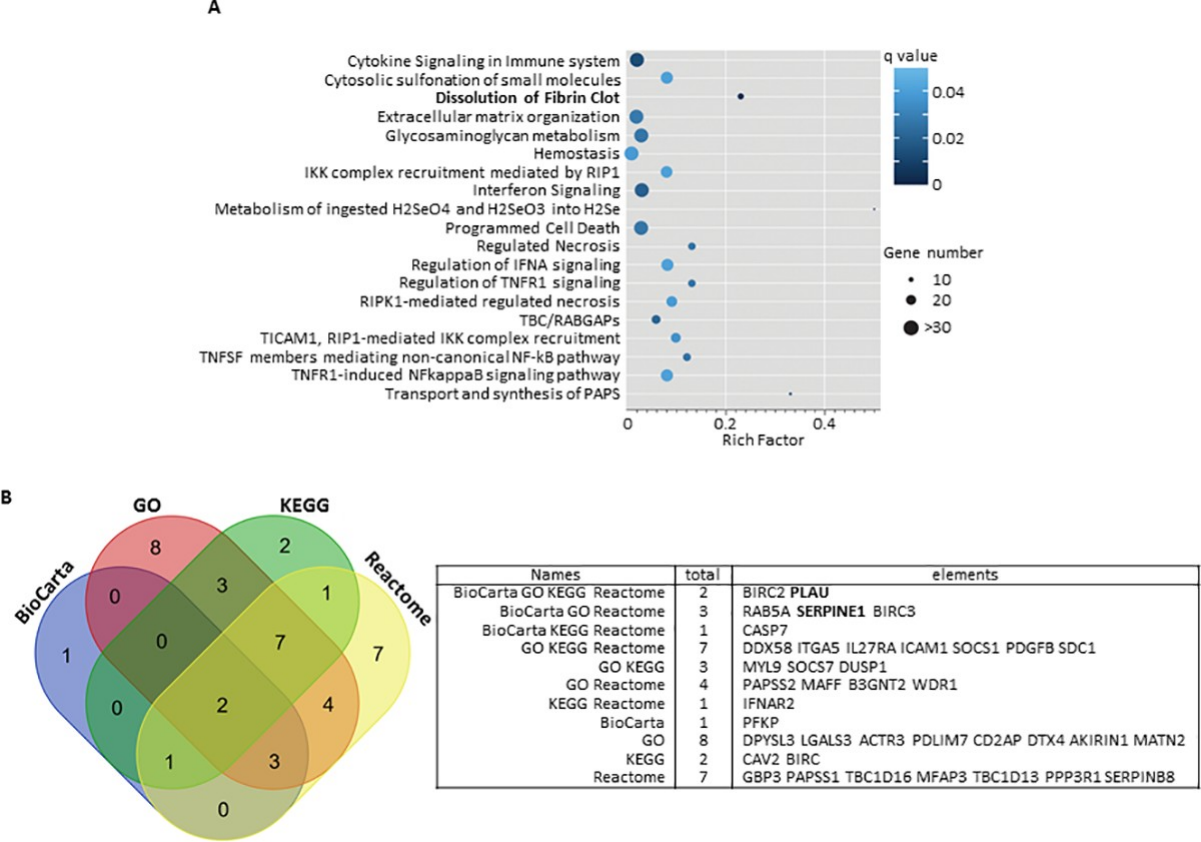
(A) Scatter plot of Reactome pathways enrichment statistics. Rich factor is the ratio of the differentially expressed gene number to the total gene number in a certain pathway. q value is corrected p value. The color and size of the dots represent the range of the q value and the number of differently expressed genes mapped to the indicated pathways, respectively. (B) Venn diagram showing the overlap of genes of the enriched biological pathways identified using BioCarta, GO, KEGG and Reactome pathway databases (S4 Table).

Pathway analysis was then applied to the top 100 most significantly differentiated mRNA expressions according to *MAST2* silencing to assess whether this genes list could be enriched for genes belonging to specific biological pathways. Enrichment analysis was performed using the DAVID software [9] interrogating the GO, KEGG, REACTOME, and PANTHER databases. At a FDR of 1%, very few pathways were identified (Fig 4A and S4 Table). Nevertheless, one of these pathways was significantly enriched (FDR = 0.002) for genes belonging to the fibrinolysis cascade notably *SERPINE1*, *PLAU* and *SERPINB8*.

Because pathway structure and terminology vary across databases, we determined which genes were significantly contributing to the overall pathway signals detected. A comparison of the significant differently expressed genes from significant BioCarta, GO, KEGG and Reactome pathways converged on two genes (*PLAU* and *BIRC2* Fig 4B). *BIRC2* codes for a E3 ubiquitin-protein ligase (Baculoviral IAP Repeat Containing 2 protein) involved in many biological processes including apoptosis and response to inflammatory and virus stimuli. A direct role of this protein in coagulation, if any, has never been documented. Conversely, *PLAU* codes for the plasminogen activator Urokinase a key molecule of the fibrinolytic pathway [9]. We thus measured plasma UPA levels in the family members and observed that they were similar in cases and controls (Fig 2). Interestingly, *SERPINE1* was found at the intersection of significant BioCarta, GO and Reactome pathways (Fig 4B). Plasminogen activation potential assessed in plasma by the global fibrinolysis assay ECLT was significantly lower in cases than in controls (1200 +/- 300 Min vs 458 +/- 90 Min, p = 0.01) reinforcing the potential alteration the fibrinolysis cascade (Fig 2).

### Impact of MAST2 Arg89Gln variant on the regulation of *TFPI* and *SERPINE1* gene expression

**Fig 5:**
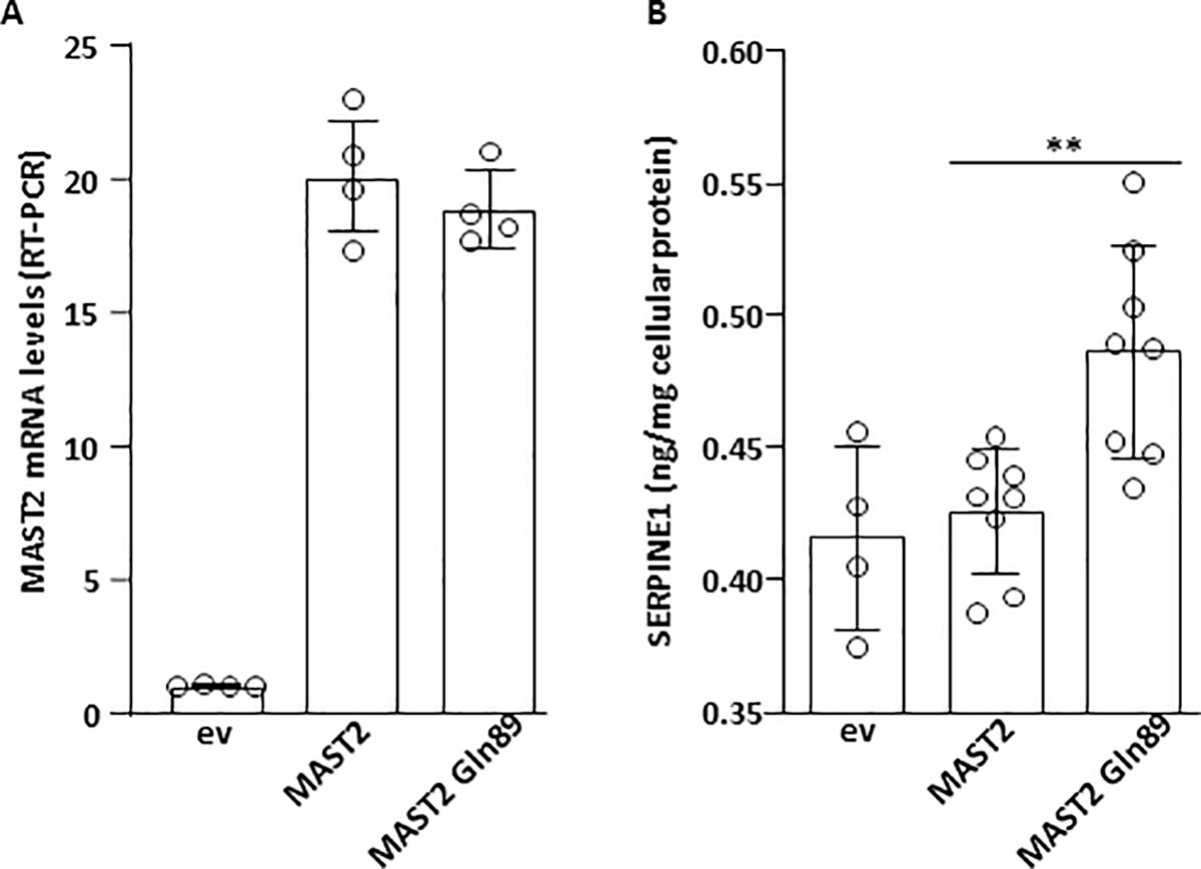
Effect of MAST2 overexpression on PAI-1 production. ECV304 cells were transfected with empty vector (ev), wild type MAST2 or MAST2 Gln89 expression vector. (A) MAST2 mRNA levels were measured by RT-PCR and expressed relative to ev transfected cells. (B) PAI-1 accumulated in the culture media over a period of 36 hours was measured by ELISA and expressed relative to the amount of protein contained in the cell lysate. Data are means ± Standard Deviation. Statistical analyses were made using t-test. **p<0.01.

**Fig 6:**
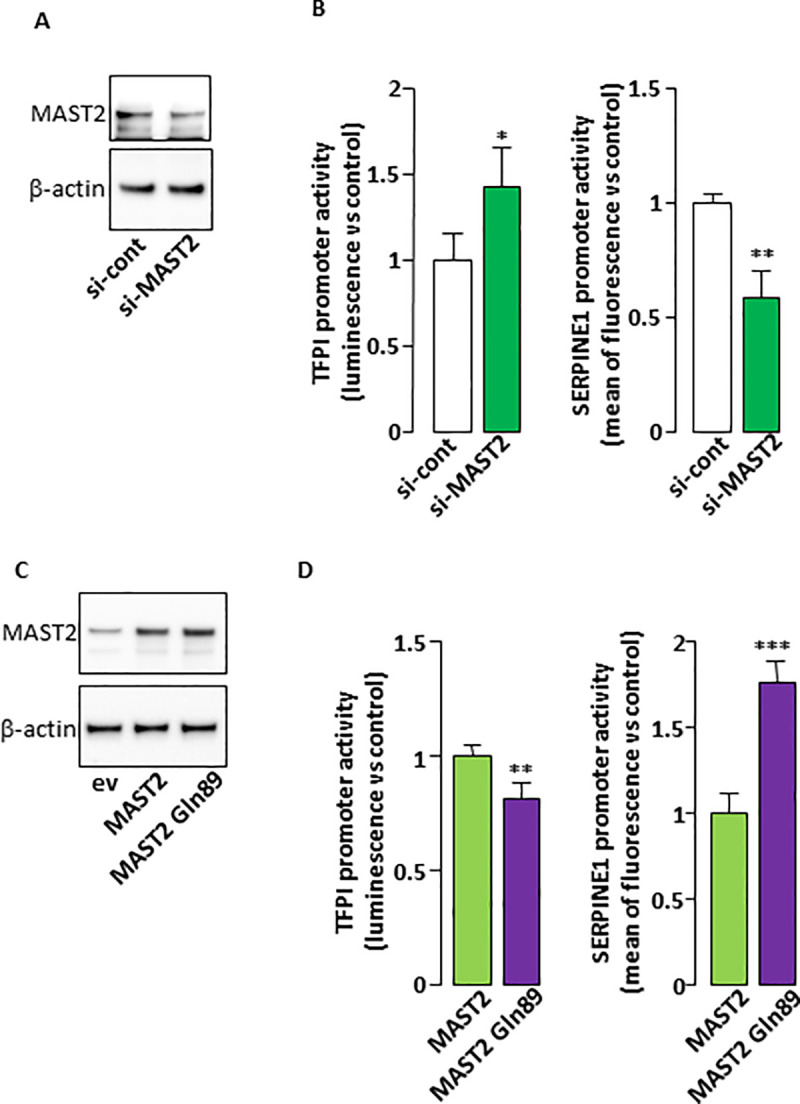
MAST2 regulates *TFPI* and *SERPINE1* promoter activity. (A). HEK293 cells were transfected with control siRNA or with the two MAST2 specific siRNA and MAST2 expression was analyzed by western blot 48 hours post transfection. The detection of β-actin attests for equal protein loading. (B). HEK293 cells were cotransfected with control siRNA or with the two MAST2 specific siRNA together with *TFPI* (left) or *SERPINE1* (right) promoter reporter. 48 hours post transfection, the activity of the promoters was measured as described in Materials and Methods section. (C). HEK293 cells were transfected with empty vector (ev), wild type MAST2 or MAST2 Gln89 expression vector and MAST2 expression was analyzed by western blot 48 hours post transfection. The detection of β-actin attests for equal protein loading. (D). HEK293 cells were cotransfected wild type MAST2 or MAST2 Gln89 expression vector together with *TFPI* (left) or *SERPINE1* (right) promoter reporter then the activity of the promoters was measured as in Fig 4B. Data are means ± Standard Deviation. Statistical analyses were made using Mann-Whitney. **p<0.01; ***p<0.001: significant vs control.

Alterations in plasma levels of PAI-1 and TFPI measured in carriers of the MAST2 Arg89Gln variant (Fig 2) associated with the regulatory role of MAST2 on the expression of the *TFPI* and *SERPINE1* genes (Fig 3C) led us to hypothesize an effect of the MAST2 Arg89Gln variant on the expression of these genes. To test this hypothesis, the expression of *SERPINE1* and *TFPI* was measured in ECV304 cells transfected with expression vector for wild type MAST2 or MAST2 Gln89 (Fig 5A). We observed that more PAI-1 accumulated (over a period of 36h) in the conditioned medium from ECV304 cells overexpressing MAST2 Gln89 than in the medium from cells overexpressing wild-type MAST2 (Fig 5B). TFPI concentrations measured in ECV304 cells conditioned media were close to the low detection limit of our ELISA assay, which prevented us from showing any effect of MAST2 overexpression on TFPI expression. Forty-eight hours after transfection of MAST2 and MAST2 Gln89, RT-PCR analysis showed no alterations in the mRNA levels of *SERPINE1* and *TFPI*. It is important to mention that at best 50% of ECV304 cells were transiently transfected, this “relatively low” transfection rate could be responsible for our inability to detect the effect of the over-expressed proteins on *SERPINE1* and *TFPI* mRNA levels. Therefore, MAST2-dependent transcriptional regulation of *TFPI* and *SERPINE1* expression was studied in HEK293 cells expressing firefly luciferase under the control of *TFPI* promoter (TFPI-Luc) or destabilized EGFP under the control of *SERPINE1* promoter (PAI-1-dEGFP). Transfection of MAST2 specific siRNA, which moderately reduced MAST2 protein levels as observed in Fig 6A, significantly increased *TFPI* promoter activity and reduced that of *SERPINE1* (Fig 6B), showing the ability of MAST2 to regulate the transcription of these genes. To investigate the effect of MAST2 Arg89Gln variant on *TFPI* and *SERPINE1* transcription, wild type MAST2 and MAST2 Gln89 were overexpressed in HEK293 cells (Fig 6C) and their impact on the activity of *TFPI* and *SERPINE1* promoter was analyzed. *TFPI* promoter activity was lower and that of *SERPINE1* higher in cells overexpressing MAST2 Gln89 than in cells overexpressing wild type MAST2 (Fig 6D). This result suggests that the transcription of the *TFPI* and *SERPINE1* genes is altered in carriers of the MAST2 Arg89Gln variant.

### Impact of MAST Arg89Gln variant of AKT phosphorylation

**Fig 7:**
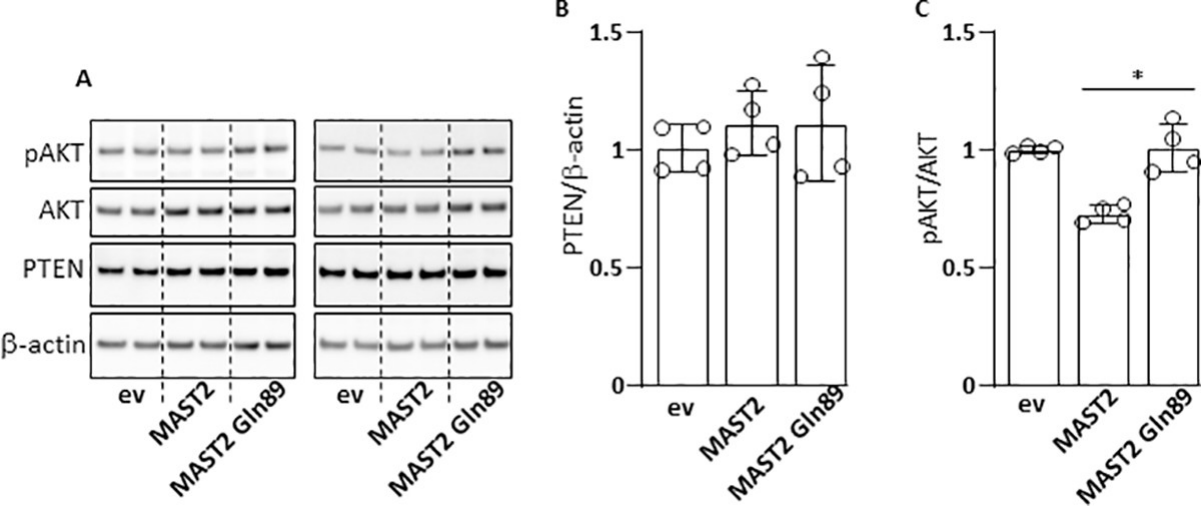
Effect of MAST2 on pAKT. ECV304 cells were transfected with empty vector (ev), wild type MAST2 or MAST2 Gln89 expression vector. 48 h post transfection cells were lysed then, AKT, phosphoAKT (pAKT), PTEN and β-actin (loading control) were detected by immunoblots (A). Chemiluminescent signals were analyzed by densitometry and ratios PTEN/β-actin (B) and pAKT/AKT (C) were calculated and expressed relative to ev transfected cells. Data are means ± Standard Deviation. Statistical analyses were made using Mann Whitney test. *p<0.05.

There is evidence in the literature for possible connections between MAST2 and phosphatase and tensin homologue deleted on chromosome 10 (PTEN)[10]. Since PTEN acts as a negative regulator of PI3K/AKT signaling, we explored the effect of overexpressed MAST2 and MAST2 Gln89 (Fig 5A) on AKT phosphorylation. Total amount of cellular PTEN was similar whether wild type MAST2 or MAST2 Gln89 was overexpressed (Fig 7A and 7B). However, we detected more phosphorylated AKT Ser473 when MAST2 Gln89 was overexpressed (Fig 7A–7C), suggesting that PI3K/AKT signaling may be affected by the MAST2 Arg89Gln variant.

## Discussion

Using a WES approach, we identified the *MAST2* Arg89Gln variant as the disease causing variant responsible for inherited thrombophilia in an extended family of French origin.

The identification of this variant, located in exon2 of the *MAST2* gene, lead us to demonstrate for the first time that this gene is an important player of endothelium in VTE by regulating key coagulation and fibrinolysis parameters. This mutation, with allele frequency of ~0,0004% in public genome/exome genomics resources, was not identified in a collection of ~6,790 VTE cases and 5,970 controls specifically genotyped for. According to the ACMG classification [11], this variation satisfies PP1 and PP3 criteria and would be classified as likely pathogenic. The identified mutation maps to the N-terminal domain of the MAST2 protein belonging to the Microtubule-associated serine/threonine-protein kinase (MAST) family. It is located in a highly conserved region across species and is predicted to be damaging by several prediction tools. The members of the MAST kinases family are characterized by the presence of a serine-threonine kinase domain, a second 3’ MAST domain with some similarity to kinase domains and a PDZ domain [12]. They were demonstrated to link and phosphorylate the dystrophin/utrophin (DMD/UTRN) network with microtubule filaments *via* the syntrophins, modulating their affinities for associated proteins[13]. This poorly understood family of proteins has been implicated in several human diseases, such as neurodegeneration and breast cancer [14]. Our study is the first to report the involvement of MAST kinases in haemostasis. According to the Human Proteins Atlas and GTEx (http://www.proteinatlas.org; https://gtexportal.org), MAST2 is mainly expressed in the skeletal muscle but also in many other tissues. At the cellular level, MAST2 is mainly expressed in the cytosol with no major cell specificity.

By screening several coagulation parameters in the investigated family members, we observed that carriers of the mutation exhibited decreased TFPI plasma levels compared to non-carriers while the opposite pattern was observed for PAI-1. Those 2 molecules, which plasma levels are not affected by vitamin K antagonists, are both synthesized by endothelial cells and are major components of the coagulation/fibrinolysis process as TFPI is the primary inhibitor of the initiation of blood coagulation whereas PAI-1 is the main inhibitor of plasminogen activation. Microtubules are crucial components of the cytoskeleton which controls endothelial cell shape, migration and proliferation[15]. In plasma, decreased in f-TFPI and increased in PAI-1 levels are both associated with the risk of thrombosis [16]. According to these observations, we speculated that MAST2 might play an important role in regulating the hemostatic function of endothelial cells and that the identified variant interferes with the coagulation/fibrinolysis process affecting the risk of thrombosis. Knockdown experiments performed in ECV304 endothelial cell line followed by RNA-seq analysis confirmed that MAST2 participates to the regulatory mechanisms associated with the dissolution of fibrin clot through some mechanisms that need to be clarified. This is consistent with the altered ECLT in cases which is considered as a useful global fibrinolysis assay assessing plasminogen activation potential [17]. Nevertheless, we showed that MAST2 acts as a negative regulator of *TFPI* expression and a positive regulator of that of *SERPINE1*, *PLAU* and *RHBLD2* coding for the transmembrane protease that cleaves thrombomodulin at the cell surface[18].

It is quite difficult to transiently transfect a high percentage of EC304 cells with an expression vector. Moreover, these cells produce minute amounts of TFPI. These two features may explain why we did not detect any effect of the overexpression of wild type MAST2 or MAST2 Gln89 on the endogenous expression of TFPI. To overcome these limitations, we performed reporter gene experiments to analyze the impact of the two forms of MAST2 on *TFPI* promoter activity. *TFPI* promoter activity was lower in cells overexpressing MAST2 Gln89 than in those overexpressing wild type MAST2. This suggests that the MAST2-dependent downregulation of TFPI transcription is increased by the mutation. The amount of PAI-1 produced by ECV304 cells, that can be easily measured by ELISA in the conditioned culture media, was increased by the overexpression of MAST2 Gln89. Coherently, reporter gene experiment shows that *SERPINE1* promoter activity was higher in cells overexpressing MAST2 Gln89 than in those overexpressing wild type MAST2. These results show that MAST2 Gln89 is more efficient in stimulating *SERPINE1* expression than wild-type MAST2. Although *PLAU* expression is regulated by MAST2, the lack of significant differences in urokinase plasma levels between control individuals and patients with MAST2 Gln89 variant suggests that it is not sensitive to the Arg89Gln variant. The MAST2-dependent regulation of *RHBDL2* expression and the impact this could have on thrombomodulin shedding is currently under investigation. However, soluble thrombomodulin plasma levels were not different between control individuals and patients (4.29 +/- 0.17 ng/mL vs 9.63 +/- 3.09 ng/mL respectively, p = 0.33), suggesting that in this particular context, modification of thrombomodulin cleavage by RHBDL2 is not sufficient to affect soluble thrombomodulin plasma level.

Overall, these data reveal that MAST2 mutation is actively involved in the alterations of TFPI and PAI-1 expressions and would contribute to the pro-thrombotic state associated with this variant.

The roles of MAST2 in cell biology and/or pathophysiology remain largely unexplored. However, the relationship between MAST2 and PTEN is probably the aspect that has been most studied in the literature. It has been previously demonstrated that MAST2 can bind to and *in vitro* phosphorylate PTEN in its C-terminal tail [10,19], suggesting that PTEN may be a physiological substrate for MAST2. PTEN dephosphorylates PIP_3_ and acts as a negative regulator of PI3K/AKT signaling. It is known that C-terminal phosphorylation of PTEN decreases its phosphatase activity, therefore contributing to higher PI3K/AKT signaling [20,21]. We considered the phosphorylation of AKT as a readout of PTEN activity and we detected more phosphorylated AKT Ser473 when MAST2 Gln89 was overexpressed, suggesting that MAST2 Gln89 phosphorylates/inhibits PTEN with increased efficiency compared with wild type MAST2. Another point of view is that the MAST2/PTEN interaction allows the recruitment/stabilization of PTEN at the plasma membrane [22,23], which allows the dephosphorylation of PIP_3_ and ultimately the dephosphorylation of AKT. In this context, we hypothesized that MAST2 Gln89 would have a reduced efficiency in recruiting PTEN at the plasma membrane.

Therefore, our results show that MAST2 Gln89 is involved in the etiology of VTE likely by interfering with the haemostatic balance of endothelial cells (alteration of the of PAI-1 and TFPI expression) on the one hand and with the phosphorylation of AKT on the other. Whether MAST2 Gln89-dependent dysregulation of endothelial PTEN/PI3K/AKT axis is responsible for the altered expression of PAI-1 and TFPI and whether it is involved in the etiology of VTE, is beyond the scope of this work and deserves further investigation. However, it is important to note that the role of the PTEN/PI3K/AKT axis in VTE is not fully understood. For example, depending on the model studied, PTEN has either pro or antithrombotic properties [24,25].

The strength of the present study lies in the careful selection of the family studied. We selected relatives (across three generations) of a family with a very strong clinically prothrombotic phenotype and no detectable known inherited thrombophilia in order to increase our chance to identify new inherited thrombophilia that are likely to be rare. A special attention has been paid to identify, in this family, cases with a strong personal and certain (documented) history of VTE who are likely to have an underlying new inherited thrombophilia and controls with no coagulation abnormalities. As with any WES project, this strategy relies on the assumption that the variants to be identified reside in coding or untranslated regions, a realistic hypothesis, for at least some variants, since all thrombophilia anomalies identified so far are located in such regions. It is demonstrated that patients with unprovoked VTE at young age are more likely to have genetic risk factors of VTE. This family was also located in a geographically limited area, the Finistère department in Brittany: such families are rare, genetically poorly mixed with people outside of Brittany and more likely to share common genes from this geographical area.

Several limitations must be acknowledged: MAST2 mainly locates to the cytosol and it is then unclear how it could be directly involved in the regulation of *TFPI* and *SERPINE1* gene expressions. Moreover, out of the 15 subjects from the family studied, plasma samples were available in only 10 (3 cases and 7 controls). Further experimental studies are mandatory to decipher the underlying mechanisms. Another is about the lack of generalizability of the result. Indeed, we found no mutated patients in more than 6700 genotyped VTE patients suggesting that the mutation is private as observed with the FIX Padua [26]. However, our results pave the way for adding *MAST2* to the list of genes to be sequenced and looked for in thrombophilia families with unprovoked VTE. More importantly, our study uncovers a new regulatory pathway of *TFPI* and *SERPINE1* expression by MAST2.

In conclusion, using a whole exome sequencing approach in a large pedigree affected with VTE, we identified an extremely rare variant in the Microtubule-associated serine/threonine-protein kinase-2 (*MAST2*) gene, that is responsible for inherited thrombophilia through a not yet-fully characterized mechanism that involves modulation of the hemostatic balance of endothelial cells.

## Materials and methods

### Ethics statement

The study was approved by the Ethics Committee ‘OUEST VIII’ (n° CPP Ouest 6–584; n°Afssaps B91453-80). All individuals included in the research signed a written informed consent to participate to the research.

### Recruitment of the family

Patient recruitment and her family members have been prospectively performed since 2010 in Finistère (France). This family has been carefully selected from the index case with acute VTE and the following predefined criteria: a) VTE occurred at young age (< 50 years); b) was documented (i.e.; symptomatic proximal deep vein thrombosis and/or symptomatic pulmonary embolism objectively diagnosed according to validated criteria)[27,28]; c) was unprovoked (i.e.: VTE was not associated with clinical risk factors such as surgery, trauma or prolonged immobilization (≥72 hours) in the past 3 months, cancer in the past 2 years, chronic inflammatory illness, autoimmune disease, pregnancy, estrogen-containing pills, hormone replacement therapy); and d) with no established biological risk factors (antithrombin, protein C, protein S deficiencies, FV Leiden, prothrombin G20210A, lupus anticoagulant, anticardiolipin antibodies). This proband should also have at least two family members across three generations with a previous history of symptomatic documented VTE with the same characteristics.

All relatives (with or without history of VTE) have been invited to participate to the study excepted family members who were adopted or less than 16 years. All the participants have been asked to come to the Brest clinical center, at the CIC INSERM 1412, for clinical and biological screening of VTE. A predefined standardized form has been used to collect clinical data (demographic and clinical past history data, complete phenotype in the case of previous thrombosis, complete pedigree across three generations) and an extensive analysis of blood coagulation has been performed. Clinical characteristics of the subjects are reported in Table 2. A biobank including DNA, RNA, serum or citrate plasma samples stored at -80°C was set up for each family member.

**Table 2:** Clinical characteristics of the cases (i.e.; with VTE) in family members.

Cases number	Gender	Relationship	WES	Age at VTE (years)	VTE type
305000	Female	Index case	Selected for WES	34	PE
305001	Female	Mother	Selected for WES	27	DVT
				45	PE
				54	PE
305002	Male	Maternal uncle	Selected for WES	32	DVT
				36	PE + DVT
305003	Female	Maternal aunt	Selected for WES	43	PE + DVT
305012	Female	Maternal	Selected for replication	35	PE

PE: Pulmonary Embolism; DVT: deep vein thrombosis

### Measurement of coagulation and fibrinolytic traits

Several coagulation and fibrinolytic parameters, not belonging to the classical thrombophilia screening, were measured in 10 family members with plasma available (3 cases and 7 controls). The 3 cases were on warfarin whereas none of the controls were on anticoagulant treatment at the time of the sampling.

Activated partial thrombin time (aPTT) and prothrombin time (PT), fibrinogen, factor II, factor V and factor X were measured in plasma with automated coagulometers (STA-Revolution, Diagnostica Stago, Asnières, France) using established commercial assays. Free tissue factor pathway inhibitor (f-TFPI) plasma levels were measured with the Asserachrom Free TFPI enzyme immunoassay from Diagnostica Stago (Asnieres, France) and plasma PAI-1 activity with the Zymutest PAI-1 activity from Hyphen Biomed (Neuville-sur-Oise, France). Urokinase (uPA) plasma levels were measured with the uPA ELISA kit (Abcam, Cambrige, UK). The euglobulin clot lysis time (ECLT) assay has been performed as previously described [29].

### DNA analysis

Fifteen family members were studied (see genealogical tree in Fig 1). Relatives with a previous VTE event as defined by our inclusion criteria were considered as cases. From 5 cases with DNA available, 4 were selected for being whole exome sequenced. These were the most distant VTE relatives. From the remaining family members who have not had any previous episode of venous or arterial thrombosis and that presented with normal thrombin generation test and a normal leg doppler ultrasound (individuals considered as controls), we selected for WES two that were expected to be as genetically close as possible from cases (Fig 1). Clinical characteristics of the WES participant are shown in Table 2.

A 2 × 100 paired-end sequencing was performed on an Illumina HiSeq2000 instrument at the IGBMC platform (http://www.igbmc.fr/).

### Bioinformatic WES workflow

FastQ sequences were aligned to the hs37d5 version of the human reference genome hg37 with BWA-MEM algorithm [30] of the Burrows-Wheeler Aligner.

All duplicated reads were marked using picard-tools-v1.119 (http://broadinstitute.github.io/picard/) and sorted using samtools-v1.3.1[31]. Base quality check was performed prior to the variant calling with GATK—BaseRecalibrator (GenomeAnalysisTK-v3.3–0).

Single nucleotide variants (SNVs) and small insertions/deletions (INDELs) were called following GATK’s Best Practices (https://software.broadinstitute.org/gatk/best-practices) with HaplotypeCaller. After the GATK’s VQSR step, «PASS» variants were annotated using ANNOVAR[32] package.

As a strategy to identify the culprit variant, we first prioritized variants that were likely functional (stop loss/stop gain, frameshift insertion/deletion, non-synonymous and splicing variants) and that were carried by all VTE cases and not by any of the two controls. Second, a bioinformatics search in public genomic data repositories (eg dbSNP, NCBI, Ensembl, 1000 genomes, GnomAD (https://gnomad.broadinstitute.org/) and FrEx (http://lysine.univ-brest.fr/FrExAC/) was carried out on prioritized variants to select as suggestive candidates those that have not been reported, or reported to be at low frequencies (<1‰). We also took into account the predicted deleteriousness of selected candidates using *in silico* tools such as SIFT, PolyPhen and CADD-v1.2 [33] to further reduce the number of candidates.

Candidate variants identified from this multi-step strategy were then genotyped in all family members for whom DNA was available, including an additional set of 1 case and 8 controls beyond the initial group of individuals that were part of the WES experiment.

### Validation of the impact of the newly identified variants in other cohorts of patients with VTE

Identified candidate variants were genotyped in additional samples of VTE patients and healthy individuals in order to get a more accurate estimation of their allele frequencies and more evidence for the putative one. Several French studies for VTE were investigated. These were:

- the “FIT study”, a prospective trans-sectional cohort assessing the risk of VTE in 2617 first-degree relatives of 507 index cases with VTE (507 families). In these families about 73% of VTE events were unprovoked. Description of the cohort has been previously reported [34].

- the MARTHA, FARIVE and EDITH case-control studies for VTE, totaling 4,173 VTE cases and 5,970 healthy individuals whose detailed description has already been reported elsewhere [5]. Percentages of unprovoked VTE events were 27%, 29% and 100% in MARTHA, FARIVE and EDITH respectively.

We retrieved information about variant rs1387081220 from large scale genotyping projects which, in aggregate, included information from 345,939 genetically independent individuals, with 60.26%, 12.51% and 12.35% being of Caucasoid, Asian and African ethnicity groups respectively. A single copy of the minor allele of rs1387081220 was observed in two female individuals from the gnomAD dataset (one Caucasian and one African) but was unobserved in the remaining 345,937 individuals across all collections. However, no phenotype information could be obtained from the gnomAD participants. The results of the look-up of genotypes is summarised in S2A and S2B Table.

### Functional characterization of the newly identified genetic variants

The role of MAST2 in endothelial function was assessed by small interference RNA (siRNA) silencing. Comparison of the endothelial transcriptome of ECV304 cells cotransfected with two MAST2 siRNA leading to a significant downregulation of MAST2 expression (around 80% reduction of mRNA levels) compared to ECV304 cells transfected with control siRNA was performed using RNA-Seq.

Total RNA extracted from transfected ECV304 cells with control or Mast2 siRNA were used to prepare libraries using the NEBNex Ultra II Direction RNA library prep kit for Illumina protocol according to supplied recommendations. Libraries were sequenced on an Illumina NovaSeq instrument using a paired end sequencing of 2 x 100bp. Sequencing and bioinformatics analysis of sequenced data were outsourced at the Integragen company (https://www.integragen.com/). Base calling was performed using Illumina Real Time Analysis (3.4.4) with default parameters. Fastq files were aligned to the reference Human genome hg38 with STAR [35] with the following parameters:—twopassMode Basic—outReadsUnmapped None—chimSegmentMin 12—chimJunctionOverhangMin 12 -alignSJDBoverhangMin 10—alignMatesGapMax 200000—alignIntronMax 200000 -chimSegmentReadGapMax parameter 3—alignSJstitchMismatchNmax 5–1 5 5—quantMode GeneCounts—outWigType wiggle—sjdbGTFtagExonParentGene gene_name). Reads mapping to multiple locations were removed. Gene expression were quantified using the full Gencode v31 annotation. STAR was also used to obtain the number of reads associated to each gene in the Gencode v31 database (restricted to protein-coding genes, antisense and lincRNAs). The Bioconductor *DESeq* package [36] was used to import raw counts for each sample into R statistical software and extract the corresponding count matrix. After normalizing for library size, the count matrix was normalized by the coding length of genes to compute FPKM scores (number of fragments per kilobase of exon model and millions of mapped reads).

### Plasmid constructs

Human TFPI promoter Firefly-luciferase reporter (TFPI-Luc), a pGL3-Basic vector containing a fragment of TFPI promoter from -1224 to +45, was previously described [37]. *SERPINE1* promoter (-483/+75) [38] was PCR amplified from THP1 genomic DNA then inserted in place of the CMV promoter in the pd4EGFP plasmid (Clontech) that contains the sequence coding for a destabilized version of EGFP. The resulting vector was named SERPINE1-dEGFP. GFP-tagged MAST2 expression vector (RG206492 form Origene) was used as template to PCR amplify only the full-length coding sequence of MAST2 (without the GFP coding sequence), which was then cloned into pCDNA3 vector using the In-Fusion PCR cloning system (Clontech).

### Cell culture and transfection

HEK293 cells (Griptite 293 MSR) were from Thermo Fisher Scientific. ECV304 endothelial [39] cells were from ATCC. The cells were maintained in culture as described by the manufacturers. Transfections of plasmid DNA were performed with PolyJet reagent (SignaGen Laboratories, Rockville, MD, USA). Transfections of siRNA alone or in association with plasmid DNA were performed with JetPRIME reagent (Polyplus transfection), as specified by the manufacturers. MAST2 specific siRNA (#4392420; ID: s42 and # AM51331; ID: 975) and negative control siRNA were from Thermofisher.

### Western blot

Identical amounts of total protein were heat-denatured and reduced (70°C; 10min) then submitted to SDS-PAGE separation on 4–12% gradient NuPAGE gels (Life Technologies, Saint Aubin, France) and transferred to polyvinylidene fluoride membranes. The membranes were blocked for 1 hour in 5% BSA solution and incubated with the appropriate primary and HRP-conjugated secondary antibodies (1:1,000 and 1:10,000 dilution, respectively). Immunodetections were performed using ECL reagent and image acquisition was performed by using a chemiluminescent CCD imager ImageQuant LAS 4000 (GE Healthcare, Velizy-Villacoublay, France). Densitometric analysis of the bands was performed with the ImageQuant TL software (GE Healthcare). MAST2 antibody (ab209079) was from Abcam, PTEN (138G6), AKT (C67E7), phospho-AKT Ser473 (193H12) and β-actin (13E) antibodies PTEN were from Cell Signaling Technology.

### Gene reporter experiments

Promoter activities were measured 48 hours after cell transfection. For the study of SERPINE1 promoter activity assay, HEK293 cells transfected with human SERPINE1-dEGFP were detached by incubation in PBS without calcium and magnesium and EGFP fluorescence was measured by flow cytometry (Accuri C5, BD Biosciences). SERPINE1 promoter activity was calculated as the means of fluorescence corrected for the value obtained with cells transfected with an empty vector and expressed as fold change compared to the control situation. For the study of TFPI promoter activity assay, HEK293 cells were cotransfected with human TFPI-Luc and SV40-driven Renilla luciferase coding vectors. Firefly and Renilla luciferase were measured in cell lysates using a luminometer (EnSight Multimode plate reader, Perkin Elmer). TFPI promoter activity was calculated as the ratio Firefly/Renilla luciferase and expressed as fold change compared to control.

### Real-Time PCR analysis

Total RNA was extracted using the Nucleospin RNA Kit (Macherey-Nagel, Hoerdt, France), cDNA was synthesized from 0.5 μg of RNA using M-MLV reverse transcriptase (Life Technologies, Saint Aubin, France) and used for PCR amplification. RT-PCR were performed on the LightCycler 480 instrument (Roche Applied Science, Meylan, France) using the Eva Green MasterMix (Euromedex, Souffelweyersheim, France). The comparative Ct method (2^-(ΔΔCT)^) was used to calculate the relative differences in mRNA expression. The acidic ribosomal phosphoprotein P0 was used as housekeeping gene. Primers sequences are available upon request. Changes *were* normalized to the mean of *control values*, which *were set* to 1.

### Statistical analyses

All data but RNA sequencing data were analyzed with GraphPad Prism software and individual *statistical two-sided tests used* are identified in the *figure legends*. P values ≤ 0.05 were considered statistically significant.

Differential analysis of RNA Seq data was performed using the Bioconductor *limma* package [40] on *voom*-transformed data in order to identify gene expression levels impacted by *MAST2* siRNA silencing. A False Discovery Rate (FDR) threshold of 1% was used to identify candidate mRNAs associated with *MAST2* knockdown. A functional enrichment analysis using the Database for Annotation, Visualization and Integrated Discovery software (DAVID;[41]), was performed on the most dysregulated genes to get insight into the biological pathways perturbated by *MAST2* knockdown. For this analysis, the GO, KEGG, REACTOME, and PANTHER databases were interrogated. The String database [https://string-db.org/cgi/input.pl] and the Genemania (https://genemania.org/) text mining search tools were also used to identify potential biological relationships between identified candidate mRNAs.

## Supporting information

S1 Table Genotype distribution of the *MAST 2* variant in family members. (DOCX)

S2 Table Reference genotyping datasets and genetically inferred ethnicity. (DOCX)

S3 Table Full list of differential association p-value of RNA-Seq based analysis to compare the mRNA expression profiles of ECV 304 endothelial cells, with those of *MAST2* knockdown cells. (XLSX)

S4 Table Pathway analysis applied to the top 100 most significantly differentiated mRNA expressions according to *MAST2* silencing. (XLSX)

S1 Data Data underlying Figs 2, 3C, 4, 5 and 7. (XLSX)
