# Supplementary material for: A rare coding mutation in the MAST2 gene causes venous thrombosis in a French family with unexplained thrombophilia: The Breizh MAST2 Arg89Gln variant
Source: PLoS Genet. 2021 Jan 19;17(1):e1009284. doi: 10.1371/journal.pgen.1009284 (PMC7846112; doi:10.1371/journal.pgen.1009284)
Supplement: S1 Table — (DOCX) [file pgen.1009284.s001.docx]

|  | **Status** | ***MAST2* R89Q** | **ADAMTS10 R169Q** |
| --- | --- | --- | --- |
| 305000 | case | R/Q | AG |
| 305001 | case | R/Q | AG |
| 305002 | case | R/Q | AG |
| 305003 | case | R/Q | AG |
| 305004 | control | R/R | GG |
| 350005 | control | R/R | GG |
| 305006 | control | R/R | GG |
| 305007 | control | R/R | GG |
| 305009 | control | R/R | GG |
| 305010 | control | R/R | GG |
| 305011 | control | R/R | GG |
| 305012 | case | R/Q | GG |
| 305013 | control | RR | GG |
| 305014 | control | RR | GG |
| 305015 | control | RR | GG |

**S1 Table. Genotype distribution of the *MAST 2* variant in family members**
