# Supplementary material for: A rare coding mutation in the MAST2 gene causes venous thrombosis in a French family with unexplained thrombophilia: The Breizh MAST2 Arg89Gln variant
Source: PLoS Genet. 2021 Jan 19;17(1):e1009284. doi: 10.1371/journal.pgen.1009284 (PMC7846112; doi:10.1371/journal.pgen.1009284)
Supplement: S2 Table — (DOCX) [file pgen.1009284.s002.docx]

| Genotyping Resource | Number of  individuals | Genotyping  method | rs1387081220  number of non-reference allele |
| --- | --- | --- | --- |
| 100,000 Genomes Project^1^ | 64,092 | WGS | 0 |
| 100K Genome Asia^2^ | 1,739 | WGS | 0 |
| gnomAD v2^3^ | 125,748 + 15,708 | WES + WGS | 1 |
| gnomAD v3^4^ | 71,702 | WGS | 1* |
| H3Africa^5^ | 320 | WGS | 0 |
| NIHR BioResource^6^ | 13,049 | WGS | 0 |
| TOPMed^7^ | 53,581 | WGS | 0 |
| Total | 345,939 |  |  |

**S2A Table. Reference genotyping datasets.**

Allele counts for the minor allele of variant rs1387081220 were retrieved from the respective databases. WGS, whole genome sequencing; WES, whole exome sequencing

* variant rs1387081220 failed allele-specific variant quality recalibration in this dataset

| Ethnic  Group | 100,000 Genomes Project | GenomeAsia 100K | GnomAD v2.1^9^ | GnomAD v3^9^ | H3Africa | NIHR BioResource | Total  N (%) |
| --- | --- | --- | --- | --- | --- | --- | --- |
| African (%) | 3.00 | 5.98 | 8.83^Ø^ | 29.30^Ø^ | 100.00 | 2.09 | 12.35 |
| East Asian and South Asian (%) | 8.98 | 81.71^¤^ | 17.87 | 4.31 | 0 | 7.85 | 12.51 |
| Not assigned or Mixed (%) | 9.12^†^ | 5.75^¥^ | 18.75^ß^ | 13.96^ß^ | 0 | 7.88 | 15.19 |
| Caucasian (%) | 78.90^‡^ | 6.56^§^ | 54.55^Þ^ | 52.43^Þ^ | 0 | 82.18^‡^ | 60.26 |

**S2B Table 2. Genetically inferred ethnicity**

Genetically inferred ethnicity was retrieved for all datasets, but TOPMed (data not yet available). Ethnicities are reported as percentages. The final column shows the total percentages weighted for the number of participants per study. Different genotype datasets identified different ethnic groups and sub-groups. We aggregated all the ethnic groups in main 4 groups that were common in all datasets.

† This group contains American and Not Assigned ethnic groups

‡ This group contains European ethnic group

¤ This group contains North-east Asian, South Asian and South-East Asian ethnic groups

¥ This group contains Oceanian and American ethnic groups

§ This group contains West-Eurasian ethnic group

Ø This group contains African and African American ethnic groups

ß This group contains Amish, Latino, Ashkenazi Jewish and Other ethnic groups

Þ This group contains Finnish and Non-Finnish European ethnic group

**Reference:**

1. Caulfield M, Davies J, Dennys M, et al.. The National Genomics Research and Healthcare Knowledgebase. December 2017.[doi:10.6084/m9.figshare.4530893.v5](https://doi.org/10.6084/m9.figshare.4530893.v5)
2. Wall JD, Stawiski EW, Ratan A, et al. The GenomeAsia 100K Project enables genetic discoveries across Asia. *Nature*. 2019;576(7785):106-111. doi:[10.1038/s41586-019-1793-z](https://doi.org/10.1038/s41586-019-1793-z). **PMID: 31802016**
3. Karczewski KJ, Francioli LC, Tiao G, et al. Variation across 141,456 human exomes and genomes reveals the spectrum of loss-of-function intolerance across human protein-coding genes. *bioRxiv*. August 2019:531210. doi:[10.1101/531210](https://doi.org/10.1101/531210)
4. Francioli L. gnomAD v3.0. *MacArthur Lab*. October 2019. <https://macarthurlab.org/2019/10/16/gnomad-v3-0/>. Accessed March 25, 2020.
5. Gurdasani D, Carstensen T, Tekola-Ayele F, et al. The African Genome Variation Project shapes medical genetics in Africa. *Nature*. 2015;517(7534):327-332. doi:[10.1038/nature13997](https://doi.org/10.1038/nature13997) PMID: 25470054
6. Ouwehand WH, on behalf of the NIHR BioResource and the 100,000 Genomes Project. Whole-Genome Sequencing of Rare Disease Patients in a National Healthcare System. *bioRxiv*. February 2019. doi:[10.1101/507244](https://doi.org/10.1101/507244). Accepted by Nature
7. Taliun D, Harris DN, Kessler MD, et al. Sequencing of 53,831 diverse genomes from the NHLBI TOPMed Program. *bioRxiv*. March 2019:563866. doi:[10.1101/563866](https://doi.org/10.1101/563866)
8. Whole exome sequencing and characterization of coding variation in 49,960 individuals in the UK Biobank | *bioRxiv*. doi[:10.1101/572347](https://doi.org/10.1101/572347)
9. FAQ | gnomAD. <https://gnomad.broadinstitute.org/faq>. Accessed March 25, 2020.
